# Inverse relationship between femoral lateralization and neck-shaft angle is a joint event after intramedullary nailing of per trochanteric fractures

**DOI:** 10.1038/s41598-023-38209-3

**Published:** 2023-07-07

**Authors:** Long Fang, Jian Qi, ZhengYu Wang, JiSong Liu, TingBao Zhao, YongJie Lin, Wei Hao

**Affiliations:** 1 Department of Orthopaedics and Traumatology, Shandong Provincial Third Hospital Affiliated with Shandong University, Shandong, China; 2 Department of Orthopaedics and Traumatology, 960th Hospital of PLA, Shandong, China

**Keywords:** Trauma, Radiography, Fracture repair, Geriatrics

## Abstract

This study explored the relationship between femoral lateralization and femoral neck-shaft angle after intramedullary nail (IM) fixation for per trochanteric fractures. 70 patients (AO/OTA 31A1-2) were investigated. Anteroposterior (AP) and lateral X-ray views pre- and post-operation were recorded. Patients were classified into three groups according to the position of the medial cortex of the head-neck fragment to that of the femoral shaft: being slightly superomedial (positive medial cortex support, PMCS), being smoothly contacted (neutral position, NP) or being displaced laterally (negative medial cortex support, NMCS). Patient demographics, femoral lateralization, and neck-shaft angle were measured and statistically analyzed pre- and post-operation. Functional recovery was evaluated by Harris score 3- and 6- months post-operation. All cases ultimately demonstrated radiographic fracture union. There was a tendency to have an increased neck-shaft angle (valgus alignment) in the PMCS group and increased femoral lateralization in the NP group (p < 0.05). Among those three groups, the change in femoral lateralization and neck-shaft angle was statistically different (p < 0.05). An inverse relationship between femoral lateralization and femoral neck-shaft angle was observed. Femoral lateralization increased correspondingly when the neck-shaft angle continuously decreased from the PMCS group to the NP group and then to the NMCS group, and patients in the PMCS group had better functional recovery than the other two groups (p < 0.05). Femoral lateralization was commonly produced after IM fixation for per trochanteric fractures. The fracture fixed in PMCS mode possesses the slightest change in femoral lateralization while maintaining valgus alignment of the femoral neck-shaft angle and good functional outcome, which is superior to NP or NMCS mode.

## Introduction

With the aging population expanding and life expectancy improving in China and worldwide, the incidence of osteoporotic hip fractures continues to increase accordingly. Hip fractures affect 18% of women and 6% of men globally^1^. Among these, trochanteric (per- and inter) fractures account for approximately 50% of total intracapsular and extracapsular cases^1^. It has been estimated that the global number of hip fractures will rise to 2.6 million by 2025 and 6.25 million by 2050^2^.

**Figure 1:**
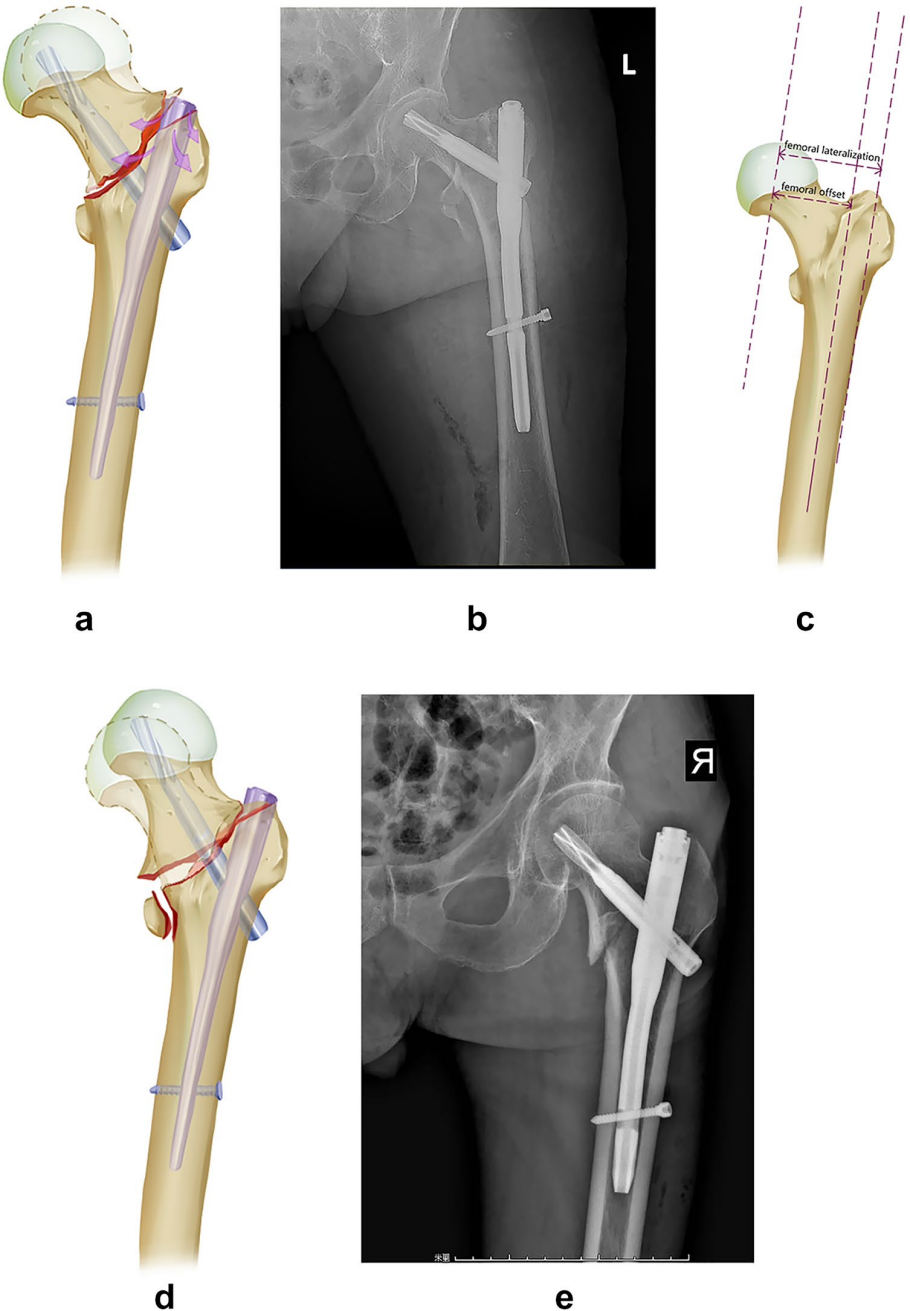
Wedge and reverse wedge effect. Wedge effect is specified as femoral shaft lateralization and varus malalignment after IM nail insertion (**a**–**c**). (**a**) Schematic diagram demonstrates occurrence of coxa vara after IM nail insertion (dotted line: anatomical position of head-neck fragment); (**b**) X ray view of wedge effect after IM nail insertion; (**c**) conception of femoral lateralization and femoral offset. Reverse wedge effect is specified as a breach at the anteromedial cortex site of fracture line with valgus alignment (**d**, **e**). (**d**) Schematic diagram demonstrates the occurrence of coxa valga after IM nail insertion (dotted line: anatomical position of head-neck fragment); (**e**) X ray view of reverse wedge effect after IM nail insertion. Notice the inferomedial gap that formed.

Intramedullary nails (IMs) have emerged as the preferred implants because of their improved biomechanical stability and reduced soft-tissue dissection^3^. Anatomical reduction is the goal of treatment but is challenging to achieve for every case in reality, primarily for unstable fracture types (AO/OTA 31A2.2, 2.3). O’Malley first observed and proposed the phenomenon of the "wedge effect," namely postoperative varus deformities(coxa vara) with the concurrence of femoral lateralization(distance of the femoral head to a line parallel to the lateral femoral cortex) after IM fixation^4^ (Fig. 1a–c). The occurrence of varus deformities post-operation leads to a higher lag screw cutout rate. Lateralized femur increases the lever arm from the center of the femoral head to the shaft, increasing axial torque imparted to the hip joint simultaneously and exacerbating the risk of internal fixator failure. Varus deformities can usually be addressed intraoperatively by abducting the injured leg. However, intraoperative correction of varus into valgus deformities is frequently accompanied by breakage of anteromedial cortices, namely the appearance of a “reverse wedge effect (coxa valga),” and consequently reduces mechanical stability (Fig. 1d, e)^5,6^. However, femoral lateralization could not be eliminated during a manual change of neck-shaft angle from wedge effect (coxa vara) to reverse wedge effect (coxa valga) intraoperation. As we proposed before^5^, the appearance of femoral lateralization maybe arise from disruption of the primary tensile trabeculae at the superolateral part of the femoral neck fragment, where it courses horizontally and perpendicularly to the longitudinal axis of the proximal femur. Due to the weaker cancellous bone structure at the great trochanter, an outward pushing force would inevitably be generated medially to laterally when the nail contacts the superolateral cortex of the femoral neck fragment during nail insertion into the medulla. Additional space is created to some extent to accommodate the proximal part of the IM nail. However, further efforts need to undertake to measure the extent of femoral lateralization, not only for varus but also for valgus deformities after IM fixation. In addition, the relationship between the change of neck-shaft angle and femoral lateralization is yet to be clarified.

This study explored the interrelationship between the femoral lateralization and neck-shaft angle after IM nail fixation of per trochanteric fractures. Considering different fracture types and various intraoperative maneuvers that surgeons usually take, we hypothesize that femoral lateralization commonly occurs and is closely related to changes in the neck-shaft angle.

## Materials and methods

The protocols in this study were approved by the Shandong Provincial Third Hospital medical ethics committee. All study methods conformed to the principles set by the Declaration of Helsinki. Informed consent was obtained from all patients and/or their legal guardians enrolled in this study for personal data collection and identification of the images in an online open-access publication.

Patients diagnosed with osteoporotic pertrochanteric fracture^7^ (AO/OTA 31A1-2) who underwent IM nail fixation at our hospital between January 2019 and December 2021 were included in this study. There were 70 male and female patients with an average age of 77.17 ± 12.08 years (Table 1) in this study. The injury mechanisms for all the patients were low-energy trauma after falling from a standing height. Exclusion criteria included contralateral hip surgery or concomitant injury to the ipsilateral femur, pathologic fractures, active malignancy, and infections. Patients were treated with Proximal Femoral Nail Antirotation II, PFNA II (Irene, China & NanXiang, China). To counteract the difficulty in having a standard patient position on the injured side during X-ray examination, anteroposterior (AP) and lateral radiographs of the contralateral side were taken along with the wounded side preoperatively to assess the personalized anatomical characteristics of the proximal femur. Then, AP and lateral radiographs of the affected hip joint were taken 2 days after the operation and compared with the radiographs of the uninjured side. The standard patient position for the AP view was supine, with the leg rotated 15°–20° medially. For lateral view, the examined side was elevated 25°–30° using a radiolucent bump to avoid overlapping the contralateral side during the examination, and the X-ray beam was horizontal.

**Table 1:** Patients’ demographics (p ≤ 0.05).

Medial cortical support effect	PMCS	NP	NMCS	p value
Age (years)	75.83 ± 13.21	79.03 ± 10.43	74.00 ± 15.87	0.507
AO/OTA classification				0.588
31A1.2	11	6	0	
31A1.3	3	3	0	
31A2.2	13	15	1	
31A2.3	9	7	2	
Total	36	31	3	
SEX				0.954
Female	26	23	2	
Male	10	8	1	
Total	36	31	3	
Alignment				0.042
Valgus	23	11	1	
Varus	13	20	2	
Total	36	31	3	
Chang reduction quality criteria: poor	0/36	0/31	1/3	0.000
Harris score				
3 months	61.32 ± 6.26	57.31 ± 9.01	49.80 ± 4.13	0.013
6 months	76.40 ± 5.57	73.27 ± 6.04	62.98 ± 2.79	0.002

**Figure 2:**
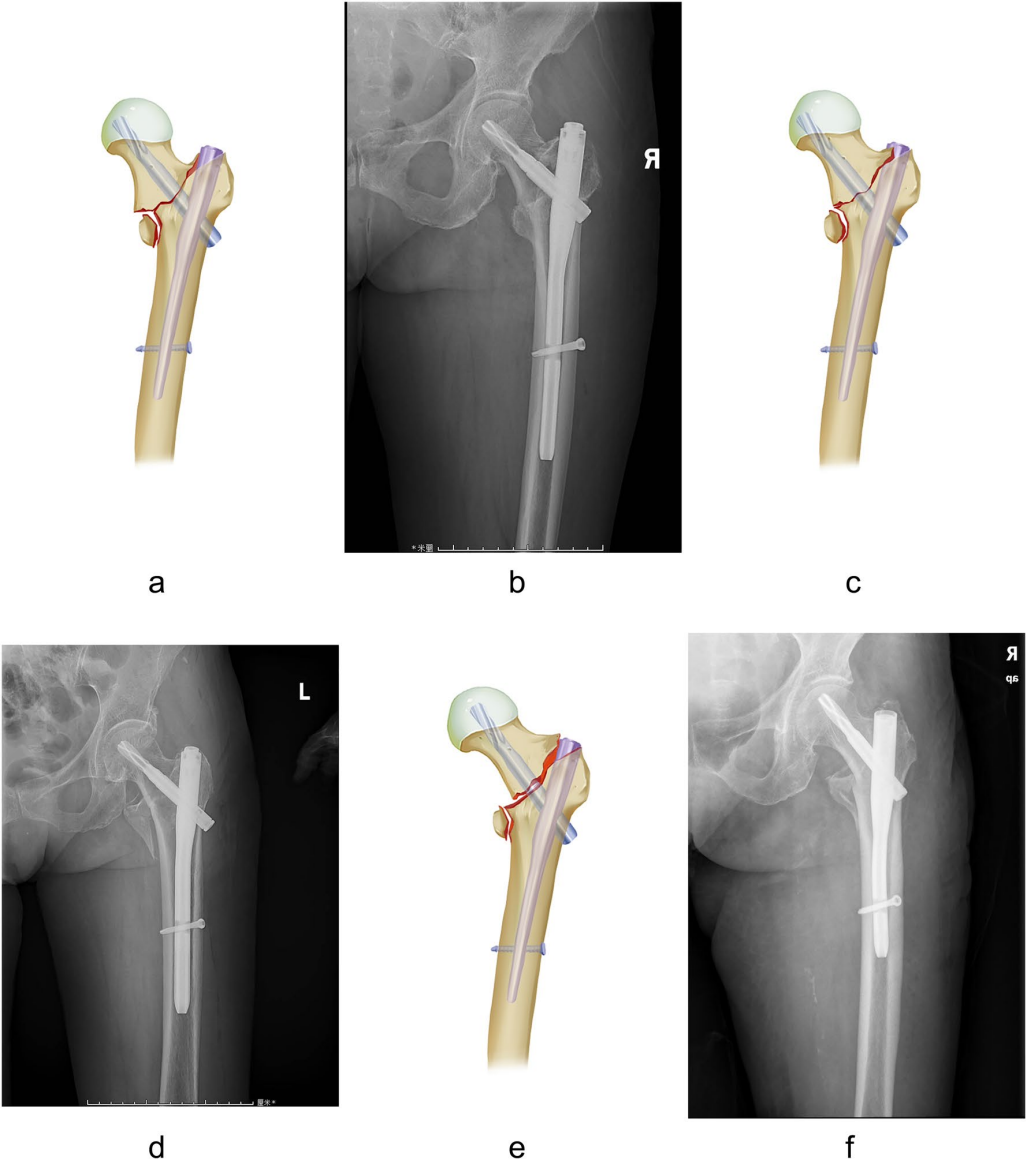
Various medical cortical support effects demonstrated as schematic diagram (**a**: PMCS, **c**: NP, **e**: NMCS) and X-ray view (**b**: PMCS, **d**: NP, **f**: NMCS).

As previously reported^8^, patients were classified into three groups according to different medial cortical support effects post-operation: (1) positive medial cortex support (PMCS): medial cortex of the head-neck fragment was located slightly (one cortical thickness, or 4–5 mm) superomedial to the upper medial edge of the femoral shaft; (2) neutral position (NP): medial cortex of head-neck and the shaft fragment is smoothly contacted; (3) negative medial cortex support (NMCS): medial cortex of the head-neck fragment is displaced laterally to the upper medial edge of the shaft fragment. Representative X-ray radiographs and related schematic diagrams are shown in Fig. 2.

After the operation, valgus or varus alignment was defined as an increased or decreased degree of a neck-shaft angle compared with that of the intact side. The degree of increase or decrease in the neck-shaft angle was recorded as positive or negative values, respectively (injured side minus contralateral side). Femoral lateralization was measured pre- and post-operation as reported before^4^: the distance of the line started from the center of the femoral head. It extended laterally until perpendicularly reaching the medial side of the lateral cortex (Fig. 1c). Compared with the intact side, increased or decreased femoral lateralization post-operation was defined as a positive or negative value, respectively (injured side minus contralateral side). Fracture reduction quality was evaluated by Chang reduction quality criteria both in AP and lateral view post-operation^8^.

The radiographs were analyzed and measured through the hospital’s imaging system (Synapse workstation, FUJIFILM systems Inc. Lexington, MA, USA.) by two experienced surgeons. Discrepancies were solved via consensus. The two surgeons were not involved in the surgery process.

Early non-weight-bearing mobilization is encouraged to start 24 h post-operation for all patients. Those activities include the full range of hip, knee, and ankle joint motion under moderate analgesics. Lower limb muscle strength exercises, including ankle pumps, knee-extension strength training, and straight leg raise, are recommended at the same time. Patients are allowed to ambulate with the walker 1-week post-surgery fixed in PMCS and NP mode and 4 weeks post-surgery fixed in NMCS mode. Physical therapists instruct and supervise patients to perform home-based exercises after discharge. Patients usually receive one-dose zoledronic acid (5 mg, intravenous route) before discharge and take calcium carbonate and vitamin D3 orally post-operation. Functional outcome was assessed 3 and 6 months postoperatively by means of the Harris hip score. Radiographs were taken for evaluation of fracture union at the same time.

### Surgical techniques

All procedures were performed by W. H. and F. L. Patients in the supine position on a fracture traction table under general or spinal anesthesia. The injured leg was routinely abducted, tracted, and internally rotated with the ipsilateral patellar kept in an anterior orientation to reduce the fracture under fluoroscopy observation both in AP and lateral view. k-wire, ball-spike pusher, or periosteal elevator would be used percutaneously or through the entry incision to facilitate reducing and temporarily maintaining reduction at fracture site during reaming and IM nail fixation.

### Interobserver reliability

Interobserver reliability for categorical variables (AO/OTA classification, medial cortical support effect) was measured using the *k* coefficient and continuous variables (Femoral neck-shaft angle, Femoral lateralization) were measured using the intraclass correlation coefficient (ICC).

### Data analysis

All analyses were performed with SPSS 22.0 (SPSS Inc., Chicago, IL, USA.). p values less than 0.05 were considered statistically significant.

The mean (standard deviation) is expressed for continuous variables. We used Fisher’s exact test (categorical variables) and one-way ANOVA (continuous variables) to test for differences among groups with various medial cortical support effects. We also used univariate linear regression analysis to further detect the connection between the femoral lateralization difference and neck-shaft angle difference among the three groups.

## Results

Among the total patients in this study, there was no significant difference concerning age, sex, or fracture classification among the three groups (PMCS, NP, and NMCS). There were 47 fractures (67%) classified as unstable (AO/ OTA -31A2). The most prevalent fracture type was AO/ OTA -31A2.2 (29 cases, 41%). The number of patients with valgus alignment (23 cases) in the PMCS group was more significant than that in the NP group (11 cases). In comparison, there were more patients with varus alignment (20 cases) in the NP group than in the PMCS group post-operation (13 cases) (p < 0.05) (Table 1). All patients demonstrated radiographic fracture union at the final 6-month follow-up. Among 70 cases in total, fraction reduction quality was evaluated as “excellent” or “acceptable” in 69 cases and “poor” in 1 case. The interobserver reliability of the radiographic variables (AO/OTA classification, medial cortical support effect, Femoral neck-shaft angle, and Femoral lateralization) is shown in Table 2.

**Table 2:** Interobserver reliability.

Variables	ICC or *k*	95%CI
AO/OTA classification (*k*)	0.939	0.872–1.007
Medial cortical support effect (*k*)	0.973	0.922–1.025
Femoral neck shaft angle (ICC)	0.909	0.872–0.935
Femoral lateralization (ICC)	0.974	0.963–0.981

Of the 70 patients enrolled, all cases (100%) were available for follow-up at 3 months and 68 (97.14%) at 6 months. By 6 months, 2 patients (2.85%) had died. Patients in the PMCS group had the best functional outcome, while patients in the NP group had the worst results at 3- and 6-months follow-up (p < 0.05) (Table 1).

In the PMCS group, the neck-shaft angle in the affected side was increased (valgus alignment) post-operation compared with the intact side (p < 0.05). At the same time, there was no statistically significant difference between the affected and intact sides in the NP and NMCS groups (Table 3). In the NP group, femoral lateralization was statistically more extended on the affected side than on the contralateral side (p < 0.05). Meanwhile, there was no difference in the PMCS and NMCS groups (p > 0.05) (Table 3). For both the difference in femoral lateralization and that of the neck-shaft angle, there were statistically significant differences that were simultaneously detected among those three groups (p < 0.05) (Table 4). In the PMCS group, the difference in femoral lateralization decreased by 4.58 mm compared with the NP group and 9.13 mm with the NMCS group. Meanwhile, the difference in the femoral neck-shaft angle in the PMCS group increased by 4.24° compared with the NP group and 6.32° with the NMCS group. In the NP group, the difference in femoral lateralization was decreased by 4.56 mm and the difference in the femoral neck-shaft angle was increased by 2.08°, compared with the NMCS group. However, significant differences relating to those two aspects were detected only between the PMCS and NP groups (p < 0.05) (Table 4) but not between the PMCS and NMCS groups or between the NP and NMCS groups (p > 0.05), which might be due to the lower number of cases (3) in the NMCS group.

**Table 3:** Analyses of Femoral lateralization and femoral neck-shaft angle (p ≤ 0.05).

Medial cortical support effect	Femoral neck-shaft angle (°)		p value
	Intact side	Injured side	
PMCS	130.36 ± 5.43	133.34 ± 6.45	0.03
NP	130.03 ± 5.81	128.77 ± 5.66	0.27
NMCS	131.43 ± 4.26	128.09 ± 6.59	0.65

Medial cortical support effect	Femoral lateralization (mm)		p value
	Intact side	Injured side	
PMCS	57.78 ± 9.06	58.41 ± 8.00	0.66
NP	57.20 ± 8.83	62.41 ± 9.31	0.00
NMCS	61.05 ± 5.86	67.49 ± 6.78	0.14

**Table 4:** Analyses of femoral lateralization difference and neck-shaft angle difference (p ≤ 0.05).

Among groups				
Medial cortical support effect	PMCS	NP	NMCS	p value
Femoral lateralization difference (mm)	0.63 ± 8.61	5.21 ± 6.85	9.77 ± 10.18	0.025
Femoral neck-shaft angle difference (°)	2.98 ± 7.91	− 1.26 ± 6.24	− 3.34 ± 10.81	0.043

Within groups				
Dependent variable	Medial cortical support effect	Medial cortical support effect	Mean difference	p value
Femoral lateralization difference (mm)	PMCS	NP	− 4.58	0.021
		NMCS	− 9.13	0.059
	NP	NMCS	− 4.56	0.345
Femoral neck-shaft angle difference (°)	PMCS	NP	4.24	0.021
		NMCS	6.32	0.155
	NP	NMCS	2.08	0.640

**Figure 3:**
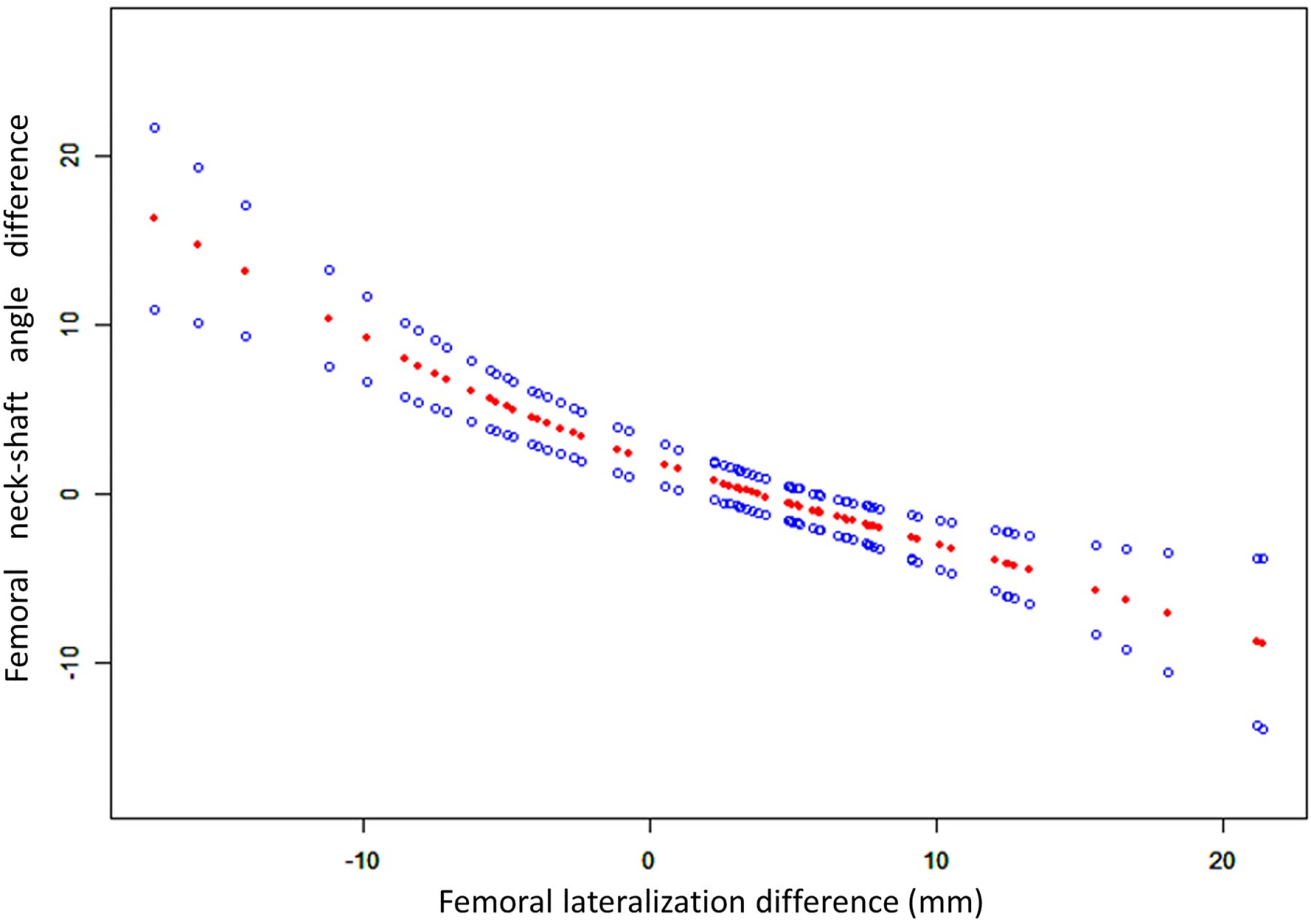
Univariate linear regression analysis. An inverse linear relationship existed between the difference in the neck-shaft angle and that of femoral lateralization.

The univariate linear regression analysis demonstrated that an inverse linear relationship existed between the difference of neck-shaft angle and femoral lateralization (p < 0.05) (Fig. 3). When the neck-shaft angle continuously decreases, femoral lateralization will increase correspondingly from the PMCS group to the NP group and then the NMCS group.

## Discussion

A previous study reported the tendency of femoral lateralization with varus deformity (wedge effect) after IM fixation^4^. We demonstrated in this report that femoral lateralization is a common phenomenon, of which the extent is closely related to various medial cortical support effects. Concerning the difference in femoral lateralization (injured side minus contralateral side), a continuously increased tendency has been observed in the PMCS group, NP group, till NMCS group. Reversely, a decreased trend concerning the difference in neck-shaft angle has been observed as well. It is indicated that the longer the femoral lateralization, the smaller the neck-shaft angle would become after IM insertion. Univariate linear regression analysis suggested that the difference in femoral lateralization is reversely related to that of neck-shaft angle.

The concept of femoral lateralization is equivalent to that of femoral offset, which is an essential measurement for characterizing hip morphology during hip arthroplasty. Femoral offset is influenced by the neck-shaft angle (NSA): it increases with varus and decreases with valgus^9^. Similarly, intentionally modifying the femoral head-neck angle in valgus alignment during IM nail fixation for per-trochanteric fracture will consequently shorten the horizontal distance between the center of the femoral head and the lateral cortex of the femoral shaft accordingly, i.e., reducing the extent of femoral lateralization, and vice versa, as proven in this study.

Femoral offset correlated positively with hip abductor strength (mainly gluteus medium muscle) and hip abduction range^10^. A report demonstrated that a decrease in the femoral offset of more than 5 mm compared to the nonimplanted side was associated with poor functional recovery, weak hip abductor muscle, gait disorders, and increased use of walking aids^11^. An increase in femoral offset might increase polyethylene wear, micromotion at the bone-implant surface, and lower 5-year survival, resulting in hip muscle pain and functional reduction^12,13^. Therefore, optimizing the femoral offset for restoring physiological hip muscle function is critical in patients with THA. Hence, femoral lateralization for per-trochanteric fracture, which can be taken as a synonym for femoral offset in THA, should be restored to its average value in each patient as much as possible. As demonstrated in this study, patients in the PMCS group exhibited minimal femoral lateralization variance while possessing better functional recovery post-operation, as compared with the NP and NMCS groups.

The intentionally nonanatomical reduction method was first proposed by Gotfried et al.^14^ during the treatment of femoral neck fracture. Generally, primary fracture stability can be accomplished by internal fixation. However, to avoid immobilization-related postoperative complications (such as pneumonia, urinary tract infections, pressure ulcers, and venous thromboembolism), early mobilization will be routinely recommended for elderly hip fracture patients with reduced activity^15^. Meanwhile, weight bearing will continuously produce stresses on the bone-screw interface and consequently may create cephalad migration of the femoral head (a tendency of "cutout") before the bone fracture heals^16^. The PMCS effect is taken as a secondary fracture stabilizer to strengthen the stability of the internal fixator^8^. In this position, better mechanical strength and lower complication rates have been achieved compared with the NP and NMCS effects^17,18^, with more favorable postoperative recovery, as shown in this study.

**Figure 4:**
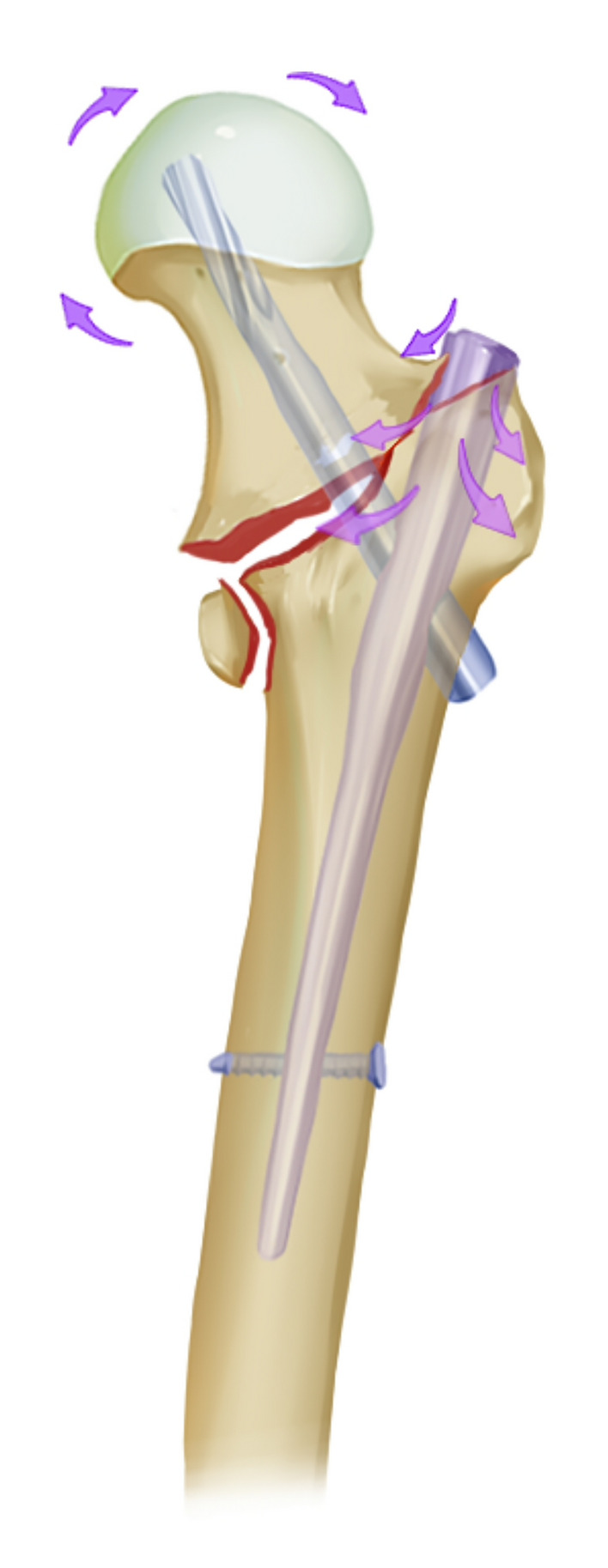
Schematic diagram demonstrating PMCS effect occurs due to internally rotated femoral head-neck fragment and femoral lateralization.

A previous study pointed out that a PMCS effect is not synonymous with the concurrence of coxa valga after IM fixation^8^. Interestingly, we demonstrated that a PMCS effect is prone to produce coxa valga, while neutral (NP) and negative medial cortical effects (NMCS) are closely related to coxa vara. As we proposed before^5^, intraoperative maneuvers play an essential role in creating these phenomena. Although provisional fracture-maintaining techniques have been used for cases that could not be well reduced through closed reduction, coincidence among the trajectory of the IM nail and fracture line make intramedullary reaming become ineffective. Bone fracture fragments during reaming would be squeezed away to some extent. This procedure will create an excessive space to accommodate the proximal part of the main nail instead of drilling a hole with an equivalent diameter to the main nail in the proximal femur. Thus, as shown in the results of this study, femoral lateralization has been proved to be a common effect among various medial cortical effects. More importantly, it suggested that femoral lateralization has no exclusive connection to either varus (such as “wedge effect”) or valgus (such as “reverse wedge effect”) deformity in neck-shaft angle after IM nail insertion. Intraoperative maneuvers, including medialization of the entry point at the greater trochanter, leverage effect acted by the proximal femur on the pivot, i.e., contact point between the superolateral cortex of the femoral neck and the proximal end of the nail, and abduction of the lower extremity, will push the proximal head-neck fragment internally rotated, i.e., into valgus alignment during IM insertion. Meanwhile, the simultaneously created outward pushing forces during IM insertion also lateralize the distal femoral shaft. Hence, internally rotated head-neck fragments with lateralized femoral shafts under continuous longitudinal traction forces would make the PMCS effect more likely to concur with valgus alignment (Fig. 4). Biomechanical testing and finite element analysis of stable and unstable intertrochanteric fractures treated with IM Nail or dynamic hip screw(DHS) suggested that a higher postreduction neck shaft angle(valgus deformity) intuitively results in a greater fracture compression force vector and a subsequently lower ratio of force causing shear at the screw-bone interface^19^, thus reduce the screw cut-out rate. IM nail and DHS possess comparable biomechanical properties^20^. The vary positions of the DHS were also analyzed, and the "valgisation" of fragments during fracture reduction was considered as positive from the biomechanical point of view. Slight valgus could reduce stress loading in the area of the femoral neck's lower edge at the fracture plane's level and shift the maximal loading point laterally to where the lag screw is located^21^.

**Figure 5:**
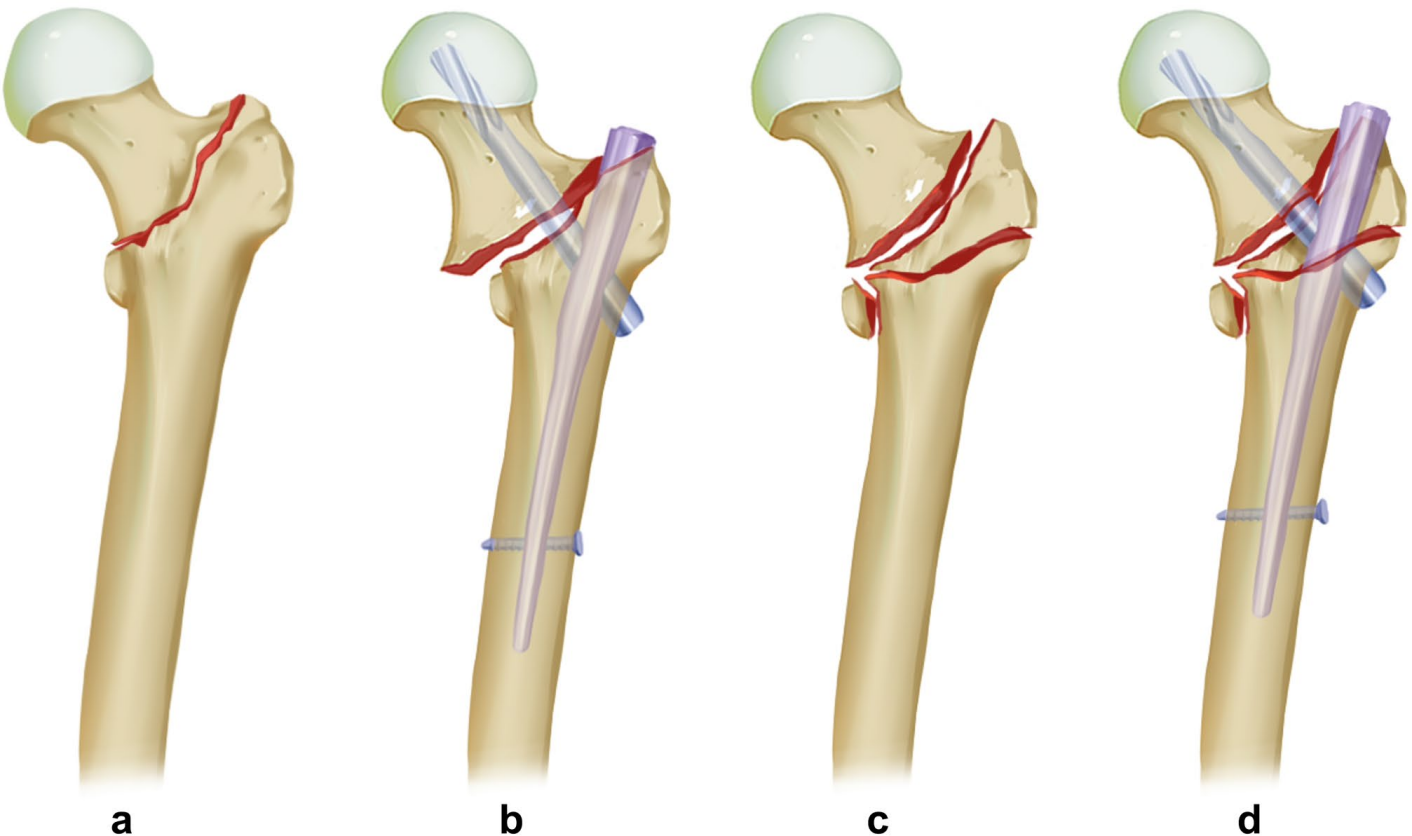
Underlying relationship of fracture stability to the occurrence of PMCS effect. (**a**) AO/OTA Fracture and Dislocation Classification 31A 1.2; (**b**) IM fixation of 31A 1.2; (**c**) 31A 2.2; (**d**) IM fixation of 31A 2.2.

Basicervical trochanteric fracture (AO/OTA 31A1.2) is predisposed to achieving the PMCS effect^6^. Although statistical analysis indicated that the incidence of the PMCS effect among various fracture types was similar in this study (p > 0.05), we found that cases that belonged to the AO/OTA 31A1.2 fracture types had the most PMCS effects post operation (11 cases in a total of 17 cases). This is closely related to the difference in fracture stability. No intermediate fragments and an intact lesser trochanter keep the 31A1.2 type the most stable fracture. This indicates that stronger outward pushing forces would be produced due to the intact superomedial and superolateral cortex around the main nail, which finally makes the femoral head-neck fragment internally rotated to a greater extent. For other fracture types (31A1.3, A2.2, A2.3), existing intermediate fragments and fractures of the lesser trochanter make the cortices around the fracture site incompetent and result in a more unstable fracture. Therefore, nail insertion-related pushing forces would be compromised and produced lower than the 31A1.2 type (Fig. 5a–d).

Some limitations exist in this study. First, there is variability in the quality of the films and techniques, which potentially influenced our measurements. Second, postoperative follow-up periods were short. Longer follow-up duration should be taken to adequately evaluate post-operative hip function. Third, nail insertion-related pushing forces have yet to be tested in this study. Further experiments will be focused on biomechanical analysis of internal fixation stability using different medial cortical support effects.

In conclusion, we demonstrated that femoral lateralization is a joint event for per-trochanteric fracture after IM nail fixation. An inverse relationship has been observed between the femoral lateralization and the neck-shaft angle. The PMCS group exhibited the slightest change in femoral lateralization while maintaining valgus alignment of the femoral neck-shaft angle. Restoring normal hip biomechanics benefits functional recovery and remains a goal for IM nail fixation of per-trochanteric fractures.

## Data Availability

We ensure datasets are deposited in publicly available repositories (WPS cloud disk/raw data, https://kdocs.cn/l/ck5tHE65jrEL). Materials described in the manuscript, including all relevant raw data, will be freely available in contact with the corresponding author to any researcher wishing to use them for non-commercial purposes without breaching participant confidentiality.
