# Author Correction: Inverse relationship between femoral lateralization and neck-shaft angle is a joint event after intramedullary nailing of per trochanteric fractures

**DOI:** 10.1038/s41598-025-08976-2

**Published:** 2025-07-15

**Authors:** Long Fang, Jian Qi, ZhengYu Wang, JiSong Liu, TingBao Zhao, YongJie Lin, Wei Hao

**Affiliations:** 1 Department of Orthopaedics and Traumatology, Shandong Provincial Third Hospital Affiliated with Shandong University, Shandong, China; 2 Department of Orthopaedics and Traumatology, 960th Hospital of PLA, Shandong, China

Correction to: *Scientific Reports* 10.1038/s41598-023-38209-3, published online 07 July 2023

The original Article contained errors in Table 1, where the p-values were incorrect for the Harris score at 3 months and 6 months due to a mislabelling that led to the initial row of data being erroneously identified as column headers and not included in the calculation of the p-values. The authors repeated the analysis including all rows of the data. The correct and incorrect values appear below.

Incorrect:

**Table:** 

Harris score	P value
3 months	0.048
6 months	0.029

Correct:

**Table:** 

Harris score	P value
3 months	0.013
6 months	0.002

The original Article has been corrected.

